# Sequential Maturation of Olfactory Sensory Neurons in the Mature Olfactory Epithelium

**DOI:** 10.1523/ENEURO.0266-19.2019

**Published:** 2019-10-14

**Authors:** Teresa Liberia, Eduardo Martin-Lopez, Sarah J. Meller, Charles A. Greer

**Affiliations:** 1 Department of Neuroscience, Yale University School of Medicine, New Haven, Connecticut 06520; 2 Department of Neurosurgery, Yale University School of Medicine, New Haven, Connecticut 06520; 3 The Interdepartmental Neuroscience Graduate Program, Yale University School of Medicine, New Haven, Connecticut 06520

**Keywords:** maturation, neurogenesis, olfactory epithelium, olfactory sensory neuron, olfactory system

## Abstract

The formation of the olfactory nerve and olfactory bulb (OB) glomeruli begins embryonically in mice. However, the development of the olfactory system continues throughout life with the addition of new olfactory sensory neurons (OSNs) in the olfactory epithelium (OE). Much attention has been given to the perinatal innervation of the OB by OSN axons, but in the young adult the process of OSN maturation and axon targeting to the OB remains controversial. To address this gap in understanding, we used BrdU to label late-born OSNs in young adult mice at postnatal day 25 (P25-born OSNs) and timed their molecular maturation following basal cell division. We show that OSNs in young adults undergo a sequential molecular development with the expression of GAP 43 (growth-associated protein 43) > AC3 (adenylyl cyclase 3) > OMP (olfactory marker protein), consecutively, in a time frame of ∼8 d. To assess OSN axon development, we implemented an *in vivo* fate-mapping strategy to label P25-born OSNs with ZsGreen. Using sampling intervals of 24 h, we demonstrate the progressive extension of OSN axons in the OE, through the foramen of the cribriform plate, and onto the surface of the OB. OSN axons reached the OB and began to target and robustly innervate specific glomeruli ∼10 d following basal cell division, a time point at which OMP expression becomes evident. Our data demonstrate a sequential process of correlated axon extension and molecular maturation that is similar to that seen in the neonate, but on a slightly longer timescale and with regional differences in the OE.

## Significance Statement

The ability to navigate using odor cues is in part dependent on the highly organized topographic map established between the olfactory epithelium and the olfactory bulb. The maintenance of this precise organization entails a challenge, since olfactory sensory neurons in the olfactory epithelium are constantly replaced throughout life. This dynamic structure combined with a fate-mapping strategy offers the ideal model to study neuronal maturation and successful axon targeting in adult neurogenesis. Our findings shed new light on the dynamics and fate of adult-born olfactory sensory neurons, which have to negotiate an established and functional system while undergoing sequential maturation and extension of their axon in a fashion that grossly recapitulates the mechanisms observed in the perinatal stages.

## Introduction

The olfactory epithelium (OE) is a pseudostratified columnar epithelium with three multicellular compartments (for review, see Sokpor et al., 2018). Progenitor cells are proximal to the basal lamina, and generate new olfactory sensory neurons (OSNs). The intermediate layer includes OSNs in different stages of differentiation. Immature OSNs (iOSNs) are located deeper in the intermediate zone, proximal to the stem cells, while mature OSNs (mOSNs) are found superficially, proximal to the lumen of the nasal cavity. The mOSNs extend an apical dendrite to the OE lumen, where it enlarges to form a dendritic knob from which 8–15 cilia extend and spread across the OE surface. OSNs are organized throughout the entire OE in overlapping domains according to the odor receptor (OR) they express (Ressler et al., 1993; Vassar et al., 1993; Sullivan et al., 1996; Miyamichi et al., 2005). Finally, the most apical layer of the epithelium includes non-neuronal (supporting) cells such as sustentacular cells (SUSs), microvillar cells, and the ducts of Bowman’s glands (Schwob et al., 2017).

The OE of juvenile mice includes ∼5,200,000 OSNs on each side of the nose (Kawagishi et al., 2014; Bressel et al., 2016), each of which expresses only 1 of ∼1200 candidate ORs (Monahan and Lomvardas, 2015). OSN axons selectively target approximately two to three glomeruli in the ipsilateral olfactory bulb (OB) based in part on the ORs that these cells express (Richard et al., 2010). OSN neurogenesis is robust throughout life (Graziadei and Graziadei, 1979; Graziadei and Monti Graziadei, 1985; Schwob, 2002; Iwema et al., 2004). This presents a challenge for the precise innervation of the glomeruli by OSN axons, as the topography of glomeruli targeting based on OR expression established during the embryonic development is maintained throughout life (Treloar et al., 2002; Cheung et al., 2014). The axons of new OSNs generated in the adult must navigate established axon tracts to innervate the appropriate glomerulus, a process that is not yet fully understood. Maintaining topography between the OSNs in the OE and their glomerular targets in the OB, despite the continuous turnover of these cells, contributes to the stability of odor representation throughout life.

The OE is dynamic and develops throughout life, and may reach a point of equilibrium in which the OSN loss is counterbalanced by ongoing OSN neurogenesis. Our understanding of OSN neurogenesis has benefited from studies that have examined different aspects of the process, including OSN survival, regeneration, proliferation, and aging (Kondo et al., 2010; Holl, 2018; Ueha et al., 2018; Savya et al., 2019). Rodriguez-Gil et al. (2015) previously characterized the maturation of perinatally generated OSNs, a period of exuberant neurogenesis that occurs before the maturation of the olfactory pathway. However, little has been done to understand the behavior of OSNs produced in a mature environment, when the olfactory system is completely formed and there is less neuronal plasticity within the resulting circuits.

In this study, we analyze the molecular maturation and axon targeting of OSNs in the adolescent mouse. We show that while the sequence of OSN molecular development is similar in perinatal and adolescent mice, neurogenesis across the OE is regionally heterogeneous. Following neurogenesis, the radial maturation of OSNs in the OE and the expression of molecular markers indicative of stages of maturation are qualitatively comparable, but with quantitative differences. Similarly, the extension of axons to the OB is slower in young adult mice compared with perinatal mice. These new data are important for understanding the dynamics of adult neurogenesis and the challenges that newly born neurons face in incorporating into the OE and extending their axons to the OB.

## Materials and Methods

### Animals

All experiments were conducted on mice produced by cross-mating Ascl1tm1.1 (Cre/ERT2)Jejo/J mice (stock #012882, The Jackson Laboratory), maintained as heterozygous (Ascl1^CreERT2/+^), with B6.Cg-Gt(ROSA)26Sortm6(CAG-ZsGreen1)Hze/J reporter mice (stock #007906, The Jackson Laboratory). Littermates with Ascl1^+/+^; R26R^ZsGreen^ genotype do not include the Ascl1-Cre/ERT2 allele and were used to assess neurogenesis rate, radial migration of OSNs, and molecular maturation. Littermates containing the Ascl1-Cre/ERT2 allele (Ascl1^CreERT2/+^; R26R^ZsGreen^) were used for analyzing OSN axonal extension. In the presence of 4OH-tamoxifen (4OH-Tx), intermediate progenitor cells express the fluorescent protein ZsGreen, allowing us to trace their lineage. Although it is not an axonal marker, ZsGreen is a reporter protein ideally suited for whole-cell labeling, including cell body and all processes. The Ascl1^CreERT2/+^; R26R^ZsGreen^ mice will subsequently be referred as double transgenic mice. All experiments randomly included both male and female mice, although sex comparisons were not pursued. Mice were housed with a 12 h light/dark cycle with access to standard food and water *ad libitum*. All animal care and use were approved by the Yale University Animal Care and Use Committee.

### BrdU and 4OH-Tx administration

For analysis of neurogenesis rate in the septal OE, Ascl1^+/+^; R26R^ZsGreen^ mice were injected with 50 mg/kg thymidine analog BrdU (BD Pharmingen) twice, 2 h apart in mice at postnatal day 7 (P7; *n* = 8) and P25 (*n* = 6). Mice were killed and assessed at 7 d post-BrdU injection (DPI-B-7). To analyze OSN migration and maturation, mice with the Ascl1^+/+^; R26R^ZsGreen^ genotype were separated into six groups (*n* = 3) and injected with BrdU (50 mg/kg) twice, 2 h apart at P25. Tissues were collected at DPI-B-1, DPI-B-3, DPI-B-5, DPI-B-8, DPI-B-10, and DPI-B-12. For analysis of the OSN axon extension, we used Ascl1^CreERT2/+^; R26R^ZsGreen^ mice exclusively at P25. These animals were distributed in 10 groups (*n* = 3) and injected with a single dose of 40 mg/kg 4OH-Tx (Sigma-Aldrich). Tissue was collected at 1, 2, 3, 4, 5, 6, 7, 8, 10, and 12 d post-4OH-Tx injection (DPI-Tx).

### Control experiments

To test the accuracy of the 4OH-Tx-inducible Cre-LoxP system, we ran three control experiments. First, we injected one group of double transgenic mice (Ascl1^CreERT2/+^; R26R^ZsGreen^) with sunflower oil (vehicle; *n* = 3) and another group with 4OH-Tx (*n* = 3). Double transgenic animals showed a considerable amount of ZsGreen^+^ OSNs in the OE at 12 d following 4OH-Tx injection (see Fig. 7*B*). However, we did not detect any specific ZsGreen^+^ neurons at 12 d post-vehicle injection (DPI-vehicle-12; see Fig. 7*C*). Likewise, we injected a third group of R26R^ZsGreen^; Ascl1^+/+^ reporter mice with 4OH-Tx, and at DPI-Tx-12 they did not show any ZsGreen^+^ OSNs in the OE (*n* = 3; see Fig. 7*D*). Using these control experiments, we established that Cre enzyme only recombines and allows ZsGreen expression in the presence of both CreERT2 gene and 4OH-Tx.

### Tissue processing

Briefly, mice were deeply anesthetized with an overdose of Euthasol (Virbac) and perfused transcardially with 0.9% NaCl in 0.1 m PBS, pH 7.4 with 1 unit/ml heparin, followed by 4% paraformaldehyde (JT Baker) in PBS. Animals were decapitated, the skin was removed from the skull and, with the brain *in situ*, was postfixed in the same fixative solution for 2 h at 4ºC, transferred to PBS overnight, decalcified in EDTA for 7–10 d at 4 C and embedded in OCT (optimal cutting temperature) compound (Thermo Fisher Scientific). Finally, 25 μm sections, including the OE and OB, were serially collected in the coronal and sagittal planes using a Reichert Frigocut Cryostat E-2800. Sections were frozen at −20ºC until use.

### Immunohistochemistry

Sections were thawed at 37ºC and treated for antigen retrieval with 0.01 m citrate buffer at pH 6 and 70ºC for 30 min. Those sections containing the thymidine analog were also treated with 0.02 m HCl at 65ºC for 30 min for DNA denature. Then, they were incubated in a blocking solution of PBS supplemented with 0.1% Triton X-100 (Sigma-Aldrich), 5% normal donkey serum (Accurate Chemicals), and 0.1% bovine serum albumin (Sigma-Aldrich) for 1 h at room temperature. Then, sections were incubated in a mixture of primary antibodies diluted in PBS with 10% blocking solution at 4ºC overnight (Table 1) followed by incubation with secondary antibodies diluted in PBS for 2 h at room temperature (Table 1). Nuclei were counterstained by incubating the section with 1 μg/ml DAPI (Invitrogen) and 5 μm DRAQ5 (BD Pharmingen). After each step, the sections were carefully washed in PBS. Finally, the sections were mounted with Mowiol 4-88 (Sigma-Aldrich). When immunostaining involved ZsGreen, antibody antigen retrieval was not performed. Please see Table 1 for details about primary and secondary antibodies.

**Table 1:** Primary and secondary antibodies used in the study

Anti	Host	Isotype	Dilution	Company (catalog #)
Primary antibodies				
AC3	Rabbit	Polyclonal IgG	1:300	Thermo Fisher Scientific (PA5-35382)
BrdU	Rat	Monoclonal IgG	1:300	Abcam (ab6326)
BrdU	Rat	Monoclonal IgG	1:300	Accurate Chemical (OBT 0030)
cCasp3	Rabbit	Polyclonal IgG	1:500	Cell Signaling Technology (9661)
GAP 43	Rabbit	Polyclonal IgG	1:1000	Millipore-Chemicon (AB5220)
Ki67	Rabbit	Polyclonal IgG	1:200	Novus (110-89719)
OMP	Goat	Polyclonal IgG	1:1000	Wako (544-10001)
ZsGreen	Rabbit	Polyclonal IgG	1:300	Clontech (632474)
Anti	Host	Label	Dilution	Company
Secondary antibodies				
Goat	Donkey	A555	1:1000	Thermo Fisher Scientific
Goat	Donkey	A647	1:1000	Thermo Fisher Scientific
Rat	Donkey	A488	1:1000	Thermo Fisher Scientific
Rabbit	Donkey	A488	1:1000	Thermo Fisher Scientific
Rabbit	Donkey	A555	1:1000	Thermo Fisher Scientific

### Imaging and quantification

BrdU images were obtained with an Olympus camera attached to an Olympus BX51 epifluorescence microscope using a 20× objective lens. For each age group (P7, *n* = 8; P25, *n* = 6), BrdU^+^ cells were manually quantified from two anatomic locations—dorsal and ventral—of the septal OE in three coronal sections evenly spaced 25%, 50%, and 75% along the anterior–posterior axis for each animal. Cell counts were performed in every image using Fiji software and represented as linear density per millimeter of septal OE. As has been previously reported (Mazzotti et al., 1998; Muñoz-Velasco et al., 2013), we recognized different patterns of BrdU labeling during the S phase of the cell cycle. During early S phase, BrdU is associated with dispersed chromatin domains far from the nuclear envelope, revealing a labeling dispersed throughout the nuclear space. However, during late S phase, BrdU labeling is found in perinuclear heterochromatin regions, revealing a ring-like labeling pattern. Both patterns of BrdU labeling were included in our analyses.

Analyses of Ki67-labeled cells were performed on 20× confocal images (LSM 800, Zeiss), *z*-stacks were 10–12 μm in depth. Ki67^+^ cells at DPI-B-1, DPI-B-3, DPI-B-5, and DPI-B-7 (*n* = 3 each) were counted using Fiji software from both anatomic locations (dorsal and ventral OE) in five coronal sections separated by 750 μm along the anterior–posterior axis.

To analyze the radial migration of BrdU^+^ cells in the OE, images were obtained with an Olympus BX51 epifluorescence microscope using a 20× objective lens. Three coronal sections evenly spaced along the anterior–posterior axis for each animal were analyzed at DPI-B-1, DPI-B-3, DPI-B-5, DPI-B-8, DPI-B-10, and DPI-B-12 (*n* = 3 each). To calculate the relative position of BrdU^+^ cells along the OE thickness, we assigned the value 0 to the lamina basal and 1 to the surface of the OE. Then, we calculated the regression line for each image and determined the relative position of every OSN. Finally, to determine accurately the position of each BrdU^+^ cell regardless of the overall height of the OE, we measured the distance between the cell and the basal lamina using Fiji software.

The molecular maturation of OSNs was studied using double immunohistochemistry with BrdU and markers of OSN maturation [Table 1: growth-associated protein 43 (GAP 43), olfactory marker protein (OMP), adenylyl cyclase 3 (AC3)]. Images of BrdU-labeled cells coexpressing one or two different markers were obtained using a 40× confocal lens (LSM 800, Zeiss) and studied on 1 µm sections collected in *z*-stacks spanning 7–9 μm in depth. For every *z*-stack, each BrdU^+^ nucleus was scanned throughout the 7–9 µm depth to confirm with certainty that the BrdU^+^ nucleus falls perfectly with the OSN expressing each specific marker (GAP 43/AC3/OMP). Three coronal sections evenly spaced along the anterior–posterior axis for each animal were analyzed for colocalization at DPI-B-1, DPI-B-3, DPI-B-5, DPI-B-8, DPI-B-10, and DPI-B-12 using Fiji software. For a more realistic interpretation, all double-immunolabeled cells were normalized to the total number of BrdU^+^ cells counted per region. Data are represented as the percentage of the average among the three animals per time point ± SEM. For confocal colocalization analyses and quantifications, a total of 305 BrdU^+^ OSNs were counted and analyzed at DPI-B-1, 388 at DPI-B-3, 360 at DPI-B-5, 185 at DPI-B-8, 206 at DPI-B-10, and 171 at DPI-B-12.

To study the axonal extension, ZsGreen-labeling was analyzed on 20x and 40x confocal images of 25-μm-thick sagittal sections using a Zeiss LSM 800. For all images, image contrast and brightness were manipulated only for display purposes using Adobe Photoshop CS6.

### Experimental design and statistical analysis

A total of 32 Ascl1^+/+^; R26R^ZsGreen^ mice were injected with BrdU, and 30 Ascl1^CreERT2/+^; R26R^ZsGreen^ mice were injected with 4OH-Tx. For each experiment, animals were injected at different ages and perfused at specific time points for *post hoc* immunohistochemical analysis, as outlined above in the BrdU and 4OH-Tx administration subsection. Multiple sections containing the OE were evaluated per animal, as described in the Imaging and quantification subsection. The resulting data from each analysis were evaluated to apply the appropriate statistical analysis. All the statistical tests were performed using Prism 7 software (GraphPad Software). Every cell count, migration, and maturation analysis were performed blind to the area and time BrdU/4OH-Tx postinjection. Differences among groups were assessed by one-way ANOVA or two-way repeated-measures ANOVA (with Bonferroni’s *post hoc* test), when appropriate. Individual tests for each experiment are specified in detail in the Results, figure legends, and Table 2. All measures across different sections within an animal were averaged to yield one sample replicate for statistical analysis. For all statistics, the number of animals in each group was represented as “*n*.” Data are shown as the mean ± SEM. The significance level was set as *p* < 0.05.

**Table 2:** Summary of statistical analysis used in each experiment

Experiment	Test	*p* Value	Statistical value	*Post hoc* test	Pairwise comparison	*p* Value
Figure 2*C* Num. BrdU^+^ (age × Anat. location)	Two-way RM ANOVA	Anat. Location = 0.0014Age < 0.0001Interaction = 0.003	Anat. Location, *F*_(1,12)_ = 17.19Age *F*_(1,12)_ = 36.21Interaction *F*_(1,12)_ = 13.28	Bonferroni’s correction	Ventral*:* P7 vs P25Dorsal: P7 vs P25	0.0002<0.0001
					P7: dorsal vs ventralP25: dorsal vs ventral	n.s.0.0005
Figure 3*E*Num. BrdU^+^ at P25(Anat. location × time point)	Two-way RM ANOVA	Anat. Location < 0.0001Time point = n.s.Interaction = n.s.	Anat. Location *F*_(1,8)_ = 134.7Time point *F*_(3,8)_ = 1.947Interaction *F*_(3,8)_ = 1.133	Bonferroni’s correction	Dorsal vs ventral:DPI 1DPI 3DPI 5DPI 7	0.00950.00060.00080.0019
Figure 3*F*Num. Ki67^+^ at P25 (Anat. location × time point)	Two-way RM ANOVA	Anat. Location < 0.0001Time point = 0.004Interaction = n.s.	Anat. Location *F*_(1,8)_ = 149.4Time point *F*_(3,8)_ = 10.06Interaction *F*_(3,8)_ = 2.666	Bonferroni’s correction	Dorsal vs ventral:DPI 1DPI 3DPI 5DPI 7	0.00140.00010.00890.0018
					Ventral:DPI 3 vs DPI 5	0.0154
Figure 3*G*Ventral Num. BrdU^+^ vs Ki67^+^ (marker × time point)	Two-way RM ANOVA	Marker = n.s.Time point = n.s.Interaction = n.s.	Marker *F*_(1,8)_ = 0.8985Time point *F*_(3,8)_ = 2.71Interaction *F*_(3,8)_ = 3.345			
Figure 4*A*Migration P25(Anat. location × time point)	Two-way RM ANOVA	Anat. Location = 0.0002Time point < 0.0001Interaction = n.s.	Anat. Location *F*_(1,8)_ = 43.85Time point *F*_(3,8)_ = 51.44Interaction *F*_(3,8)_ = 3.477	Bonferroni’s correction	Dorsal vs ventral:DPI 3DPI 8	0.02120.0026
					Dorsal:DPI 1 vs DPI 3Ventral:DPI 1 vs DPI 3DPI 5 vs DPI 8	0.027 <0.00010.0025
Figure 4*B*Migration P25(Anat. location × time point)	Two-way RM ANOVA	Anat. Location = 0.0001Time point = 0.0103Interaction = 0.106	Anat. Location *F*_(1,8)_ = 50.03Time point *F*_(3,8)_ = 7.511Interaction *F*_(3,8)_ = 2.826	Bonferroni’s correction	Dorsal vs ventral:DPI 4DPI 6DPI 8	0.01440.02540.0033
Figure 4*C*Ventral migration P25 (time point)	One-way ANOVA	<0.0001	*F*_(5,12)_ = 30.28	Bonferroni’s correction		n.s. individual comparisons
Figure 4*E*OSNs maturation (marker × time point)	Two-way RM ANOVA	BrdU/marker = 0.0067Time point < 0.0001Interaction < 0.0001	Marker *F*_(2,24)_ = 6.22Time point *F*_(5,12)_ = 32.45Interaction *F*_(10,24)_ = 13.85	Bonferroni’s correction	Marker dynamic:GAP 43:DPI 3 vs DPI 5DPI 10 vs DPI 12AC 3:DPI 5 vs DPI 8DPI 8 vs DPI 10OMP:DPI 8 vs DPI 10DPI 10 vs DPI 12	0.02340.0011 0.040.0168 0.01510.00031
					Differential marker expression at each time point:DPI 5:GAP 43 vs AC 3GAP 43 vs OMPDPI 8:OMP vs AC 3OMP vs GAP 43DPI 10:AC 3 vs OMPDPI 12:GAP 43 vs AC 3GAP 43 vs OMP	0.0004<0.0001 0.0050.0075 0.0055 <0.0001<0.0001

Anat. location, Anatomic location; num., number; RM, repeated measures; n.s., not significant.

## Results

### Neurogenesis in the Septal OE at P7 versus P25

**Figure 1.**
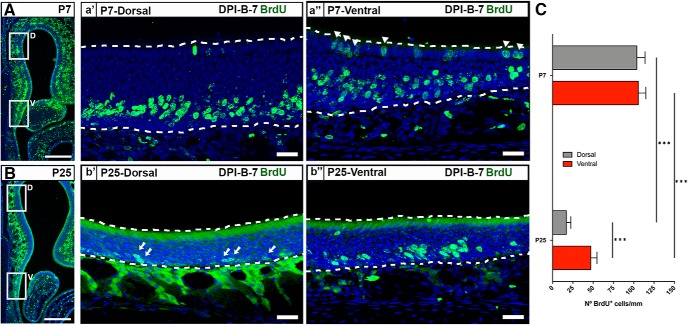
Neurogenesis in the septal OE at P7 and P25. ***A***, P7-born OSNs are evenly distributed in the dorsal and ventral zones of the septal OE. ***a′***, ***a″***, BrdU labeling (green) at DPI-B-7 in the dorsal (***a′***) and ventral (***a″***) domains of the septum at P7. ***B***, P25-born OSNs are predominant in the ventral zone of the septal OE. ***b′***, ***b″***, Representative image of BrdU labeling (green) at DPI-B-7 in the dorsal (***b′***) and ventral (***b″***) domains of the septum at P25. Note the low density of BrdU^+^ cells in the dorsal OE at P25 (arrows). ***C***, Histograms show the number of BrdU^+^ cells in dorsal and ventral zone of the septal OE at P7 (*n* = 8) and P25 (*n* = 6). The mean ± SEM values are plotted. ***p < 0.001. Dotted white lines delineate OE luminal surface (top) and basal lamina (bottom). DRAQ-5 is in blue. Scale bars: ***A***, ***B***, 300 μm; ***a′***, ***a″***, ***b′***, ***b″***, 25 μm.

To analyze the dynamics of OSN postnatal neurogenesis, we used BrdU pulse labeling at two different ages: P7 and P25. Following a 7 d survival period, we quantified BrdU labeling in the OE of the dorsal and ventral septum. BrdU labeling was most prominent superior to the basal lamina and was largely limited to the somatic domain of the OSNs (Fig. 1*A*,*B*). Scarce labeling at the OE surface, proximal to the lumen of the nasal cavity, reflected ongoing SUS genesis and was not further analyzed (Fig 1*a*″, arrowhead). Labeling deep to the basal lamina in connective tissue and secretory cells was similarly not included in our analyses.

Labeling of newly generated OSN nuclei was robust throughout the OE at both ages (Fig. 1). When comparing the density of BrdU^+^ cells in the septal wall, we found a significant main effect of both factors analyzed, as follows: age (*p* < 0.001) and anatomic location (*p* < 0.01; Fig. 1*C*). The number of BrdU^+^ cells in the ventral septal OE in P7 mice was 2.2 times higher than that in P25 mice (105.6 ± 9.2 at P7 vs 47.03 ± 7.7 at P25, *p* < 0.001; Table 2) and 6 times higher in the dorsal septal OE (103.7 ± 10.2 at P7 vs 17.1 ± 5.4 at P25, *p* < 0.0001; Table 2). Likewise, there was a significant age-related distribution of BrdU^+^ cells along the dorsal–ventral axis of the OE. In P7 mice, BrdU^+^ cells were evenly distributed in both dorsal and ventral septal OE (Fig. 1*A*,*a*′,*a*″,*C*). However, at P25, they were highly clustered in the ventral septum (Fig. 1*B*,*b*′,*b*″,*C*). These results are consistent with an age × location interaction in the postnatal proliferation of OSNs.

**Figure 2.**
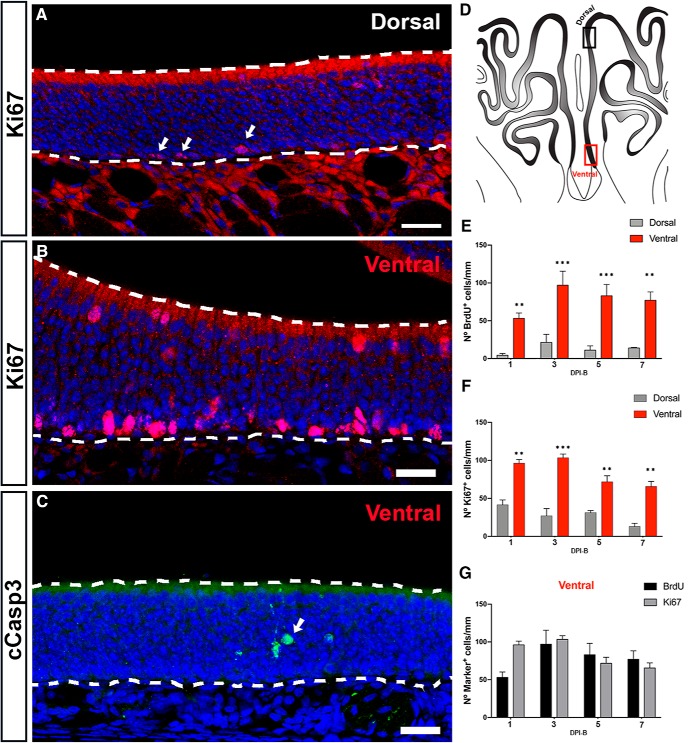
Cell proliferation in the septal wall of the OE in P25 mice. ***A***, ***B***, Maximum intensity projections of confocal *z*-stacks showing Ki67-expressing cells (red) in the dorsal (***A***) and ventral (***B***) domains of the septum. Note the low density of Ki67^+^ cells in the dorsal zone (arrows). Ki67-labeled cells at the luminal surface of the ventral septum are sustentacular cells. ***C***, Confocal image showing cCasp3-expressing cells in the ventral septal OE (green). Note the low number of apoptotic cells (arrow). ***D***, Illustration of the OE from a coronal perspective. Black and red rectangles represent dorsal and ventral zones of the septal OE, respectively. ***E***, ***F***, Histograms show the number of BrdU^+^ cells per millimeter (***E***) and Ki67^+^ cells per millimeter (***F***) in the dorsal and ventral zones of the septal OE at different following time points after BrdU injections: DPI-B-1, DPI-B-3, DPI-B-5, and DPI-B-7. The mean ± SEM values are plotted (*n* = 3). ***G***, Histogram representing the number of BrdU^+^ and Ki67^+^ cells in the ventral septal OE. The mean ± SEM values are plotted. **p < 0.01, ***p < 0.001. Dashed white lines delineate the surface of the OE (top) and basal lamina (bottom). DRAQ-5 is in blue. Scale bars, 25 μm.

We then turned our attention to the process of OSN genesis in the septal OE at P25 and investigated the cellular mechanisms underlying the scarce number of P25-born BrdU^+^ cells in the dorsal septal OE. First, we assessed whether the number of BrdU^+^ cells underwent significant changes over time depending on their anatomic location. We analyzed the density of BrdU^+^ cells in the dorsal and ventral septal OE 1, 3, 5, and 7 d following BrdU injections. A two-way ANOVA showed a main effect of anatomic location (*p* < 0.0001), but no effect of time postinjection. *Post hoc* tests showed that the number of BrdU^+^ cells was significantly higher in the ventral area when compared with the dorsal at each time point (Fig. 2*E*). Of interest, in both the dorsal and ventral septum BrdU labeling peaked at 3 DPI-B, but did not show a statistically significant effect between the time points that were analyzed when tested with two-way ANOVA (Fig. 2*E*) (cf. Suzuki and Takeda, 1993; Vedin et al., 2009; Kondo et al., 2010; Savya et al., 2019). Given that the total number of cells is determined by the balance between proliferation rate and cell death, we performed immunostaining for cleaved-caspase-3 (cCasp3), a marker for apoptotic cells, and for Ki67, a cellular marker for proliferation (Fig. 2*A–C*). We focused on Ki67^+^ cells located close to the basal lamina of the OE (which give rise to future OSNs) and did not study Ki67^+^ cells located in the superficial lamina of the OE that belong to the SUS population. A two-way ANOVA showed a main effect of anatomic location for Ki67^+^ cells (Fig. 2*A*,*B*,*F*; *p* < 0.0001). However, in contrast to the BrdU data, we also found a significant main effect of time for Ki67 (*p* < 0.01). *Post hoc* multiple pairwise comparisons established that the number of proliferating neurons significantly decreased from DPI-B-3 to DPI-B-5 in the ventral septal OE. Given that the probability of a type I error is higher when repeated measures of two-way ANOVAs are applied, we next compared the number of BrdU^+^ and Ki67^+^ cells at different time points. Statistical analyses did not show a significant effect of time on the number of BrdU^+^ and Ki67^+^ cells in the ventral septal OE (Fig. 2*G*; *p* = n.s.), supporting the idea of a possible type I error (Table 2, for statistical analysis applied and *p* values). These results are consistent with prior work demonstrating that cells expressing Ascl1, a transcription factor in nascent OSNs, are few in number in the dorsomedial part of the OE in 8-week-old mice, but not in 1-week-old mice (Vedin et al., 2009).

**Figure 3.**
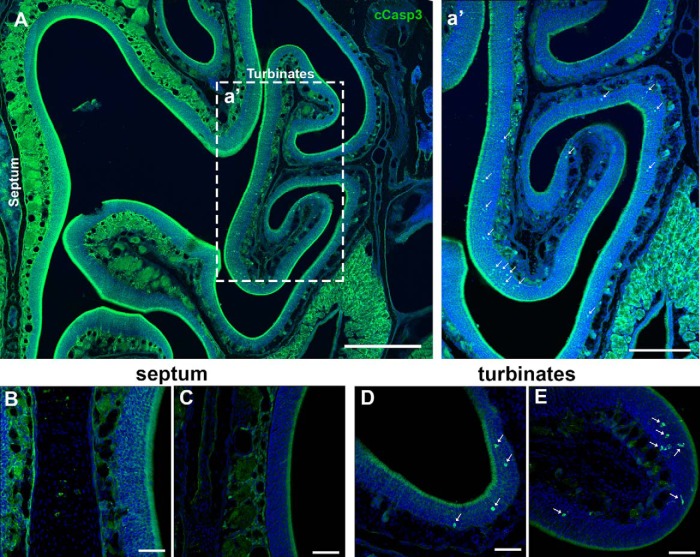
Cell death in the OE in P25 mice. ***A***, Semipanoramic view of the OE including the septum and turbinates. ***a′***, Caspase 3^+^ cells (green) are mainly spread across the turbinates (arrows). Almost no caspase 3^+^ cells were observed along the septal wall. ***B***, ***C***, High magnification of the septal wall showing the absence of caspase 3^+^ cells. ***D***, ***E***, High magnification of different turbinates showing the presence of several caspase 3^+^ cells (arrows). Scale bars: ***A***, 400 μm; ***a′***, 200 μm; ***B–E***, 50 μm.

The number of cCasp3^+^ cells in the septal OE was too small to permit statistical analysis. We observed only one to two cCasp3^+^ cells along the whole length of the bilateral septal OE in 2 of 12 animals. However, when we analyzed five sections per animal from four different time points (*n* = 3, for each time point), we observed that the presence of apoptotic cells in the turbinates was higher when compared with the septum (Figs. 2*C*, 3). These results are consistent with the notion that programmed cell death, OSN apoptosis, can vary throughout the OE.

### Differentiation and maturation of P25-born OSNs

**Figure 4.**
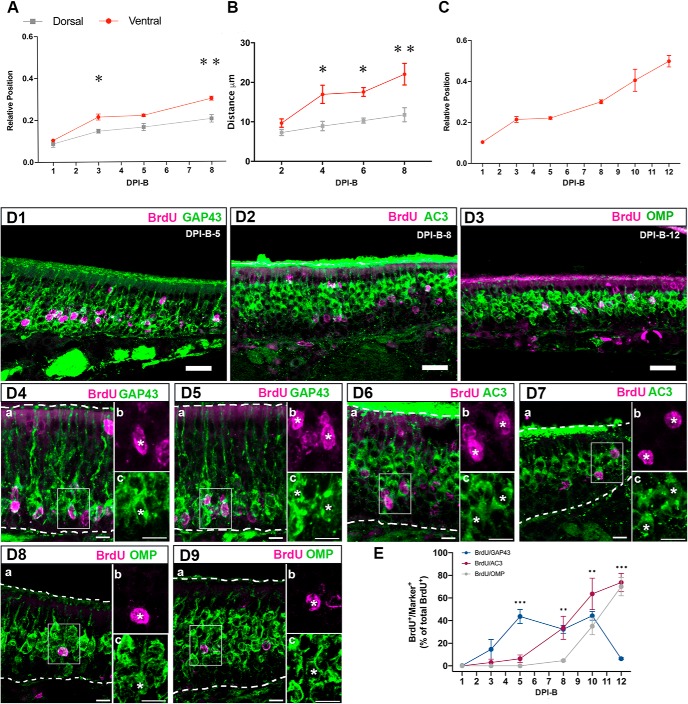
Migration and maturation of P25-born OSNs in the septal OE. ***A***, Newborn OSNs migrate radially in the OE. Graph showing relative radial position of BrdU^+^ cells in the dorsal (gray) and ventral (red) septal OE at different time points after BrdU injections. ***B***, Graph showing the distance between BrdU^+^ cells and the basal lamina; distance is represented in micrometers. ***C***, Relative position of P25-born OSNs in the ventral zone of the septum at six different time points during a 12 d timecourse after BrdU administration. The mean ± SEM values are plotted (*n* = 3). *p < 0.05, **p < 0.01. ***D***, Coimmunolabeling for BrdU (magenta) and markers of maturation (green): GAP 43, AC3, and OMP at different time points after BrdU administration. ***D1–D3***, Confocal images at low magnification representing flattened stacks of 10–12 1-μm-thick optical sections. ***D4–D9***, High-magnification confocal images representing flattened stacks of seven to nine 1-μm-thick optical sections. GAP 43 and BrdU costaining at DPI-B-5 (***D4***, ***D5***). AC3 and BrdU costaining at DPI-B-8 (***D6***, ***D7***). OMP and BrdU costaining at DPI-B-12 (***D8***, ***D9***). In ***D4a–c to D9a–c***, square cells (***a***) are magnified in ***b*** showing BrdU labeling, and in ***c*** showing the corresponding markers GAP 43, AC3, and OMP. In both ***b*** and ***c***, colabeled cells are marked with an asterisk. ***E***, Line graph showing the percentage of colabeled (marker/BrdU) cells of the total number of BrdU^+^ cells counted. Transition from immature to mature OSN occurs at 10 d after basal cell division. OSNs first express AC3 at 5 d post-basal cell division. OMP expression is not evident until 8 d post-basal cell division. The mean ± SEM values are plotted (*n* = 3). **p < 0.01, ***p < 0.001. Dashed white lines delineate the surface of the OE (top) and basal lamina (bottom). DRAQ-5 is in blue. Scale bars: ***D1–D3***, 25 μm; ***D4–D9***, 10 μm.

Newborn cells initially located close to the basal lamina undergo morphologic and molecular transitions as they differentiate into mOSNs, including radial migration into the OE. To investigate the differentiation and maturation of OSNs, we first analyzed their radial migration within the OE. Because the thickness of the OE is not equal in the dorsal and ventral regions of the septum in P25 mice, we analyzed the relative positions of OSNs along the basal–apical axis of the dorsal and ventral septal OE. We assigned the value 0 to the basal lamina and the value 1 to the surface of the OE, adjacent to the lumen of the nasal cavity. We found that P25-born OSNs are located in the lower 10% of the OE at DPI-B-1 in both dorsal and ventral regions of the septum (Fig. 4*A*). Subsequently, after DPI-B-1, neurons migrate at different rates in an anatomic location-dependent manner. P25-born OSNs are located in the lower 15% of the OE in the dorsal region of the OE between DPI-B-3 and DPI-B-5, while they reached 21% of the OE in the ventral region, suggesting a faster migration. Finally, by DPI-B-8 they reached the 20% of the OE in the dorsal region and the 30% in the ventral area of the septal OE. A two-way ANOVA comparing the relative positions of cells at different time points in the dorsal and ventral septal OE, showed a significant main effect of both of the factors time (*p* < 0.0001) and anatomic location (*p* < 0.001). These data suggest that cells migrate radially following basal cell division in both the dorsal and ventral domains of the septum, but they differ in the rate of migration depending on the anatomic location. *Post hoc* multiple pairwise comparisons displayed significantly different relative locations of cells at different time points when dorsal and ventral areas of the septal OE were compared (Fig. 4*A*; Table 2, for statistical analysis applied and *p* values). In like manner, the relative locations of cells is significantly different between DPI-B-1 and DPI-B-3 in the dorsal and ventral OE, and between DPI-B-5 and DPI-B-8 in the ventral part of the septal wall (Table 2, for statistical analysis applied and *p* values).

Because the ventral OE is thinner than the dorsal OE, the use of the ratio may not give a fully accurate assessment of migration rate. To test further that the migration rate differs in the dorsal versus ventral OE, we measured the distance (in micrometers) between the cell body and the basal lamina at different time points following the administration of BrdU. A two-way ANOVA comparing the distance traveled by BrdU^+^ cells in both anatomic locations at different time points showed a main effect of both factors time (*p* < 0.05) and anatomic location (*p* < 0.001). *Post hoc* multiple pairwise comparisons displayed significant difference in the distance traveled by cells at DPI-B-4, DPI-B-6, and DPI-B-8 when dorsal and ventral areas of the septal OE were compared (Fig. 4*B*; Table 2, for statistical analysis applied and *p* values).

Given the low number of P25-born OSNs in the dorsal septal OE, we focused on OSNs produced in the ventral region of the septum. First, we expanded the analysis of radial migration by adding two more time points to our analyses. We found that P25-born neurons reached the 40% of the OE by DPI-B-10 and that they resided in the middle of the OE by DPI-B-12. One-way ANOVA showed a significant difference in the relative positions of P25-born OSNs over time in the ventral region of the OE (*p* < 0.0001; Fig. 4*C*; Table 2, for statistical analysis applied and *p* values).

**Figure 5.**
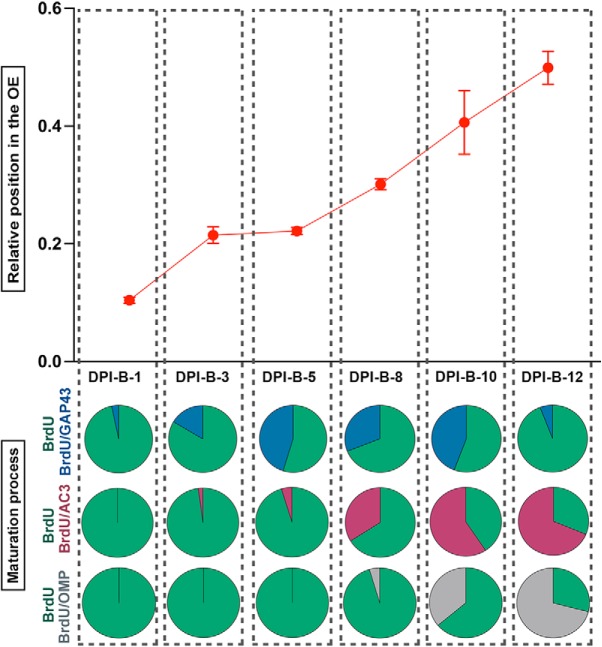
Summary correlating migration and maturation of P25-born OSNs in the ventral zone of the septal OE. Top diagram shows the relative radial position of BrdU^+^ cells throughout the OE during a 12 d timecourse following BrdU administration. Pie charts located on the bottom half of the diagram show the percentage of the following costaining cells: BrdU^+^/GAP 43^+^ (blue), BrdU^+^/AC3^+^ (red), and BrdU^+^/OMP^+^ (gray) of the total number of BrdU^+^ cells counted. BrdU^+^ cells not expressing any other marker are represented in green. Dotted vertical columns represent each time point of analysis after BrdU administration: DPI-B-1, DPI-B-3, DPI-B-5, DPI-B-8, DPI-B-10 and DPI-B-12.

Our next aim was to determine when newborn OSNs differentiate and become mOSNs. To accomplish that goal, we used BrdU pulse-chase experiments, and waited 1, 3, 5, 8, 10, and 12 d after BrdU injections to perfuse the animals, collect tissue, and carry out double-labeled immunohistochemistry to uncover the relative timing of maturation-related protein expression in P25-born cells. We used immunohistochemistry to determine the expression of the following: GAP 43 (Fig. 4*D1*,*D4*,*D5*), a protein expressed specifically in iOSNs; AC3 (Fig. 4*D2*,*D6*,*D7*), an enzyme that catalyzes the conversion of ATP to cAMP and plays a key role in axonal projection, postnatal maturation, and OR stabilization (Trinh and Storm, 2003; Chesler et al., 2007; Col et al., 2007; Zou et al., 2007; Lyons et al., 2013; Zhang et al., 2017); and olfactory marker protein (OMP; Fig. 4*D3*,*D8*,*D9*), a maturation marker in OSNs whose expression correlates with the onset of synaptogenesis (Farbman and Margolis, 1980; Monti Graziadei et al., 1980). For a more realistic comparison between BrdU^+^ cells expressing each marker at different time points, the results are presented as a percentage (Fig. 4*E*). All double-labeled cells were normalized to the total number of BrdU^+^ cells counted per linear unit (220 μm of length). Our results illustrate that BrdU^+^ cells expressing GAP 43 were rare until DPI-B-3, when 14.67% of the total number of BrdU^+^ cells were GAP 43^+^. BrdU^+^/GAP 43^+^ cells peaked at DPI-B-5, representing an average of 44% of the total BrdU^+^ cells and remained high until DPI-B-10, before they decreased abruptly at DPI-B-12 (6.33% of the total number of BrdU^+^ cells). When compared with GAP 43, OMP expression appeared significantly later. BrdU^+^/OMP^+^ cells were observed at DPI-B-8 for the first time and constituted only the 4.67% of total BrdU^+^ at that time. However, the percentage of BrdU^+^/OMP^+^ increased significantly after DPI-B-8, constituting 35% of the total number of BrdU^+^ cells at DPI-B-10, and 70% at DPI-B-12, reflecting the timescale when the expression of GAP 43 declines. In like manner, we analyzed the AC3 timeline of expression and demonstrated that it preceded OMP expression. An average of 6.33% of the total number of BrdU^+^ cells expressed AC3 at DPI-B-5, and its expression increased steadily onward. At DPI-B-8, 33.67% of the total number of BrdU^+^ cells expressed AC3, and this percentage increased to 63.67% and 73.67% at DPI-B-10 and DPI-B-12, respectively. Statistical analyses showed a significant main effect of time on each marker expression (*p* < 0.0001). Likewise, analyses showed a main effect of marker (*p* < 0.01), which means that the expression of the three markers is significantly different at each day following basal cell division. Finally, there is a significant interaction effect between the factors markers and time (*p* < 0.0001). *Post hoc* multiple pairwise comparisons showed that, chronologically, the percentage of BrdU^+^/GAP 43^+^ cells increased abruptly from DPI-B-3 to DPI-B-5 and then decreased dramatically from DPI-B-10 to DPI-B-12. In contrast, BrdU^+^/OMP^+^ cells are rare or nonexistent until DPI-B-8, but increased significantly fast during the following days (DPI-B-10 and DPI-B-12). Right before the expression of OMP, BrdU^+^ cells were also AC3^+^. The percentage of BrdU^+^/AC3^+^ cells underwent a steady increase from DPI-B-5 onward. When *post hoc* multiple pairwise comparisons were applied, the number of BrdU^+^/GAP 43^+^ cells was shown to be significantly different from the average of BrdU^+^/AC3^+^ and BrdU^+^/OMP^+^ cells at DPI-B-5 and DPI-B-12. Similarly, the number of BrdU^+^/OMP^+^ cells was significantly different from the number of BrdU^+^/GAP 43^+^ cells at DPI-B-8 and the number of BrdU^+^/AC3^+^ cells at DPI-B-8 and DPI-B-10 (Table 2, for appropriate test and *p* values). The timecourse and molecular characteristics of P25-born OSN maturation are summarized in Figure 5.

**Figure 6.**
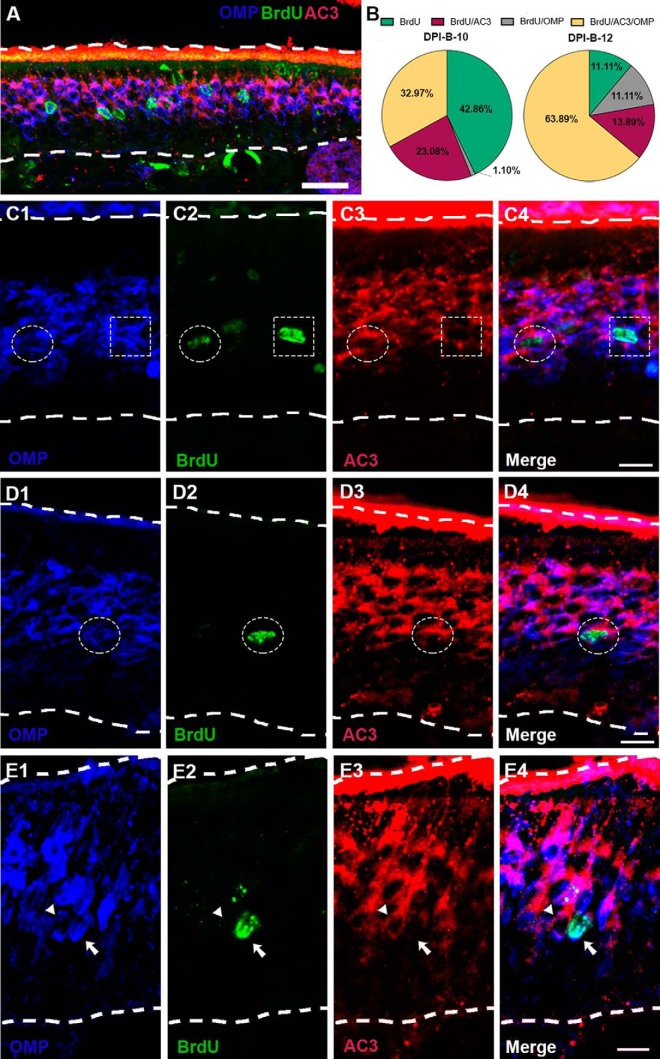
BrdU/AC3/OMP triple immunostaining at 10 and 12 d following BrdU administration. ***A***, Confocal image at low magnification representing flattened stacks of seven to nine 1-μm-thick optical sections. BrdU^+^ (green) OSNs coexpressing AC3 (red) and OMP (blue) at day 12 after BrdU injections (arrows). ***B***, Pie charts quantifying BrdU^+^ cells expressing AC3 (maroon) or OMP (gray), coexpressing AC3 and OMP (yellow), or not expressing either AC3 and/or OMP (green) at DPI-B-10 and DPI-B-12. ***C***, ***D***, Higher-magnification confocal images showing examples of BrdU^+^ cells (green) coexpressing AC3 (red) and/or OMP (blue). ***C1–C4***, Dotted circle denotes a BrdU^+^ cell (***C2***, green) coexpressing OMP (***C1***, blue) and AC3 (***C3***, red), while a dotted square highlights a BrdU^+^ cell (***C2***, green) expressing OMP (***C1***, blue), but not AC3 (***C3***, red); ***C4*** shows a merged image. ***D1–D4***, A dotted circle shows a BrdU^+^ cell (***D2***, green) coexpressing OMP (***D1***, blue) and AC3 (***D3***, red); ***D4*** shows a merged image. ***E1–E4***, Arrow pointing to a BrdU^+^ cell (***E2***, green) expressing OMP (***E1***, blue), but not AC3 (***E3***). Next to the BrdU^+^ cell, there is a BrdU^−^ cell (arrowhead) coexpressing OMP (***E1***, blue) and AC3 (***E3***, red); ***E4*** shows a merged image. Dashed white lines delineate the surface of the OE (top) and basal lamina (bottom). Scale bars: ***A***, 25 μm; ***C–E***, 10 μm.

Our results showed that P25-born OSNs start expressing AC3 immediately before they express OMP, which is interpreted as indicative of an mOSN, analogous to perinatal stages (Rodriguez-Gil et al., 2015). Functionally, AC3 is required for odor perception and successful OSN axonal projection, and is implicated in OSN maturation (Zhang et al., 2017). In light of this, and knowing that 10 and 12 d following basal cell division a significant percentage of P25-born OSNs express AC3 and OMP (Figs. 4*E*, 5), we asked whether their expression follows a sequential fashion or whether, on the contrary, there are mOSNs (OMP^+^ cells) that do not express AC3. To address this question, we conducted triple immunostaining for BrdU, AC3, and OMP (Fig. 6*A*,*C–E*). The results showed that at DPI-B-10 an average of 23.08% of total BrdU^+^ cells express AC3, only 1.1% of total BrdU^+^ were BrdU^+^/OMP^+^, and 32.97% of total BrdU^+^ cells express both markers AC3 and OMP (Fig. 6*B*). At DPI-B-12, 13.89% of total BrdU^+^ cells were also AC3^+^, and 11.11% of BrdU^+^ OSNs expressed only OMP, but not AC3 (Fig. 6*B*,*C*, dotted square, *E*, arrow). The percentage of BrdU^+^ cells expressing both markers AC3 and OMP increased to 63.89% at DPI-B-12 (Fig. 6*B*,*C*, dotted circle, 6*D*, dotted circle). These data demonstrate that most of the BrdU^+^/OMP^+^ OSNs previously expressed AC3 at DPI-B-10 and DPI-B-12, corroborating the hypothesis of sequential expression. We observed only a small percentage of BrdU^+^ cells expressing OMP, but not AC3, at DPI-B-12 (Fig. 6*B*,*C*, dotted square, 6*E*, arrow).

### Axon extension from the OE to the OB

**Figure 7.**
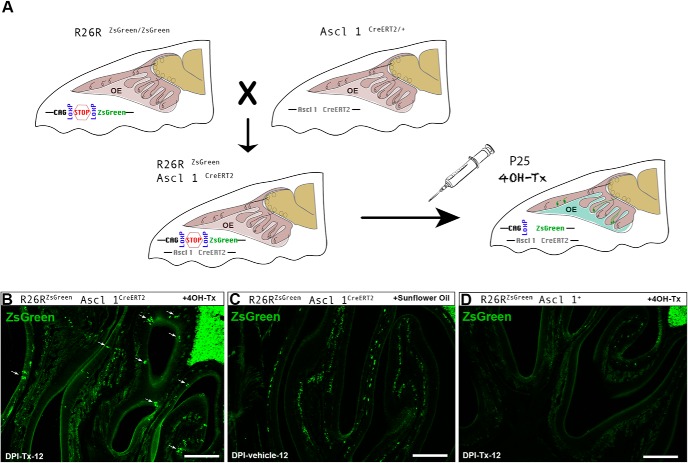
Inducible Cre-recombinase strategy for labeling P25-born OSNs in the OE following basal cell division. ***A***, Ascl1^CreERT2^/R26R^ZsGreen^ double transgenic mice were obtained by crossing an Ascl1^CreERT2/+^ transgenic mice line with an R26R^ZsGreen/ZsGreen^ transgenic mice line. 4OH-Tx induces Cre recombination in Ascl1^+^ cells, which results in the expression of ZsGreen in Ascl1^CreERT2^/R26R^ZsGreen^ double transgenic mice. ***B–D***, Cre-recombinase strategy controls: Ascl1^CreERT2^/R26R^ZsGreen^ double transgenic mice show olfactory sensory neuron-specific labeling 12 d after 4OH-Tx injection (***B***), but no specific labeling after sunflower oil injection, although some nonspecific labeling is present deep in the OE and beneath the basal lamina (***C***); Ascl1^+^/R26R^ZsGreen^ transgenic mice show no labeling after 4OH-Tx injection (***D***). Scale bars, 300 μm.

Once we analyzed the neurogenesis, radial migration, differentiation, and molecular maturation processes of the OSNs in the septal wall of the OE in young adults, we analyzed the maturation of these neurons by defining the time course and spatiotemporal features of their axon extension from the OE to the OB. To achieve this aim, we used an *in vivo* genetic fate-mapping strategy that allows us to label, in a very narrow temporal window, globose basal cells that function as transit-amplifying neuronally committed progenitor cells and express Ascl 1 (Schwob et al., 2017). We used transgenic mice that express an inducible Cre enzyme fused to estrogen receptor (i.e., CreERT2) under Ascl 1 promoter, Ascl1^CreERT2/+^ (Kim et al., 2011). Then we crossed them with R26R^ZsGreen/ZsGreen^ reporter mice to obtain the double transgenic mice used for our experiment, Ascl1^CreERT2/+^; R26R^ZsGreen^ (Fig. 7*A*). The fused protein CreERT2 stays enzymatically inactive in the cytoplasm in the absence of 4OH-Tx, but becomes transiently active after 4OH-Tx administration. In the cytoplasm, 4OH-Tx binds CreERT2 with high affinity and translocates the fusion protein into the nucleus, where Cre enzyme recombines, allowing ZsGreen expression. To test the accuracy of the model, we ran several control experiments (see Materials and Methods).

**Figure 8.**
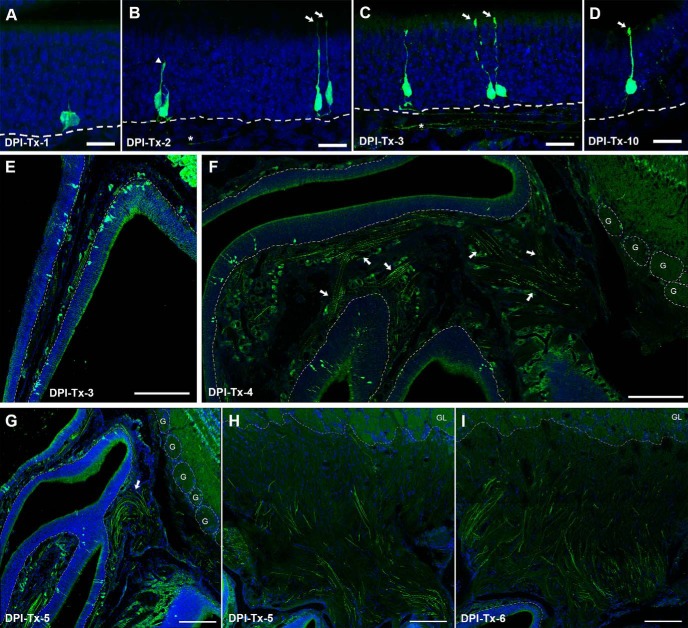
*In vivo* genetic fate-mapping strategy reveals spatiotemporal morphologic features of P25-born OSNs. ***A–D***, ZsGreen^+^ cell bodies migrate radially toward the surface of the OE during a 12 d time course following basal cell division, revealing that most of them are differentiated as OSNs with characteristic morphologic features. OSN apical dendrites and axons can be detected as early as 2 d following 4OH-Tx injection (***B–D***), but not earlier (***A***). ***E–H***, ZsGreen^+^ axons form fascicles starting at 3 d following 4OH-Tx injection (***E***) and travel toward the OB (***F***), and cross the cribriform plate at DPI-Tx-4/5 (***G***, arrow), and some of them reach the oONL as early as DPI-Tx-5 (***H***). ***I***, By DPI-Tx-6, the oONL is heavily innervated by ZsGreen^+^ axons. DRAQ-5 is in blue. Dashed white lines delineate the basal lamina in ***A–G***, and the limit between the glomerular layer (GL) and the ONL in ***H***. Dotted circles delineate glomeruli in ***G***, and each glomerulus is designated by the letter “G.” Scale bars: ***A–D***, 20 μm; ***E***, ***F***, ***H***, ***I***, 200 μm; ***G***, 300 μm.

In the same manner as the BrdU pulse chase, this model allowed us to use a different technique to analyze and confirm that P25-born somata migrated apically toward the lumen as they mature (Fig. 8*A–D*). However, contrary to BrdU strategy, the inducible Cre-LoxP system allowed us to label OSNs entirely, and we could detect ZsGreen labeling strongly expressed throughout the cytoplasm and processes of OSNs. Thus, we analyzed the spatiotemporal morphologic features of ZsGreen^+^ OSNs at 10 different time points following 4OH-Tx injection (DPI-Tx-1, -2, -3, -4, -5, -6, -7, -8, -10, and -12).

**Figure 9.**
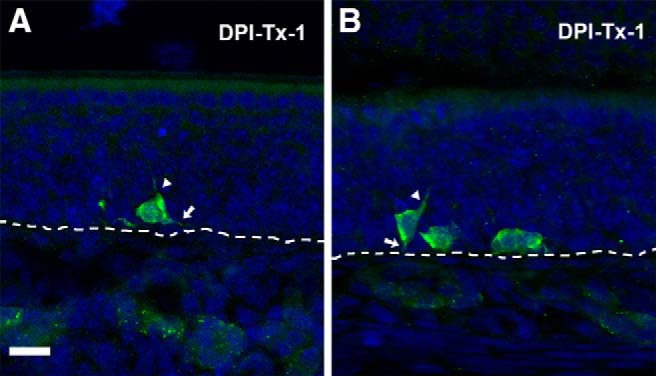
*In vivo* genetic fate-mapping strategy reveals no evidence of polarity in P25-born OSNs. ***A***, ***B***, ZsGreen^+^ cell bodies lying on the basal lamina (dashed line) at DPI-Tx-1. Initial extension of the apical dendrite (arrowhead) and axon (arrow). Scale bar, 20 μm.

**Figure 10.**
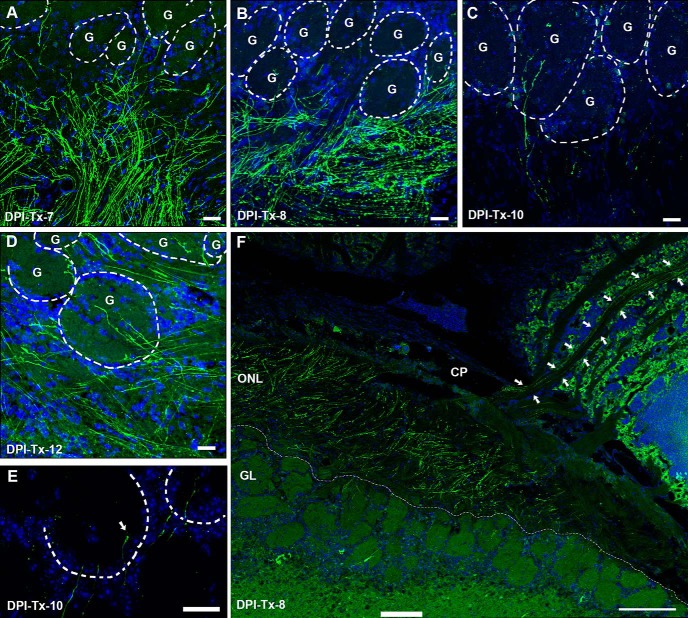
Tracking ZsGreen^+^ axon extension in Ascl1^CreERT2^/R26R^ZsGreen^ double transgenic mice at different time points following 4OH-Tx injection. ***A***, ***B***, ***F***, ZsGreen^+^ axons innervate iONL profoundly at 7-DPI-Tx (***A***), and they surround ventral glomeruli closely at DPI-Tx-8; some scarce axons enter the GL (***B***, ***F***). Panoramic sagittal view showing a ZsGreen^+^ axon pathway (arrows) from the OE to the ventrolateral OB (***F***). ***C***, ***D***, Labeled axons unequivocally innervate glomerular neuropil by 10 d following basal cell division. ***E***, Detail of ZsGreen^+^ axon growth cones (arrow) in the GL. DRAQ-5 is in blue. Dashed circles delineate glomeruli in ***A–D***, and each glomerulus is designated by the letter “G.” Dotted white lines delineate the limit between the GL and the ONL in ***F***. CP, Cribriform plate; GL, glomerular layer. Scale bars: ***A–D***, 25 μm; ***E***, 20 μm; ***F***, 200 μm.

At DPI-Tx-1, ZsGreen labeling revealed only cell bodies that were proximal to the basal lamina of the OE (Fig. 8*A*). Although scarce labeled processes were observed at this point, no evidence of polarity in the OSN was detected. In other words, both the apical dendrite and axon appear simultaneously following basal cell division (Fig. 9*A*,*B*). Noticeably, processes from OSNs were clearly distinguished by DPI-Tx-2. ZsGreen^+^ OSNs extended apical dendrites radially toward the surface of the OE, some of which reached the lumen of the nasal cavity and exhibited a dendritic knob (Fig. 8*B*, arrows). Those that did not reach the surface showed dilated elliptical tips that resembled dendritic growth cones (Fig. 8*B*, arrowhead). Within the lamina propria, the first ZsGreen^+^ axons were observed at DPI-Tx-2 (Fig. 8*B*, asterisk). At DPI-Tx-3, the vast majority of ZsGreen^+^ apical dendrites reached the lumen of the nasal cavity and showed a dendritic knob (Fig. 8*C*, arrow). Occasionally, we could distinguish some cilia extending from the knob (Fig. 8*D*, arrow). By DPI-Tx-3, numerous ZsGreen^+^ axons grouped and formed nerve fascicles that traveled toward the OB (Fig. 8*C*, asterisk, *E*). By DPI-Tx-4, the fascicles of ZsGreen^+^ axons extending from the OE to the OB (Fig. 8*F*, arrows) began to cross the cribriform plate (Fig. 8*F*). By DPI-Tx-5, after crossing the cribriform plate, ZsGreen^+^ axons joined the olfactory nerve layer (ONL) on the surface of the OB (Fig. 8*G*, arrow), where they were mainly confined to the outer ONL (oONL; Fig. 8*G*,*H*). At DPI-Tx-6, labeled axons reached the inner ONL (iONL; Fig. 8*I*) and then expanded their innervation of this layer by DPI-Tx-7 and DPI-Tx-8 (Fig. 10*A*,*B*) by surrounding olfactory glomeruli of the ventrolateral OB (Fig. 10*F*). Although occasionally a few ZsGreen^+^ axons were observed within the glomerular neuropil at DPI-Tx-8, they were clearly ramifying within the glomerular neuropil by DPI-Tx-10 (Fig. 10*C*). Moreover, this strategy also allowed us to identify several axon growth cones, revealing that those young axons are indeed pathfinding (Fig. 10*E*). We did find a noticeable decrease in ZsGreen labeling at DPI-Tx-10 and DPI-Tx-12 (Fig. 10*C*,*D*) compared with prior time points. The most parsimonious explanation for this is the occurrence of changes in the stability of the fluorescent protein ZsGreen over time.

## Discussion

**Figure 11.**
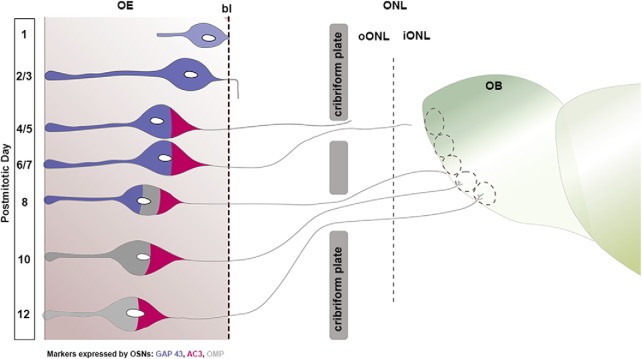
Schematic integrating three aspects of the OSN maturation process based on a compilation of postmitotic data following both the BrdU and 4OH-Tx protocols at P25: (1) cell body radial migration in the OE; (2) differential marker expression; and (3) axon extension from the OE to the OB. Cell morphology changes; expression of GAP 43 (blue), AC3 (maroon), and OMP (gray); and axonal extension progress are correlated with the corresponding postmitotic day. P25-born OSNs extend their apical dendrite and reach the OE surface 2/3 d following mitosis; simultaneously, they extend their axons deep in the basal lamina and express GAP 43; 4 and 5 d after basal cell division, axons cross the cribriform plate and reach the oONL, at which stage cells express AC3; 1/2 d later, axons innervate the oONL and iONL broadly. OSNs express OMP by postmitotic day 8, and, concurrently, their axons reach the GL, ramifying themselves into the glomerular neuropil from day 10 following basal cell division onward. Black dashed line represents the basal lamina. bl, Basal lamina; GL, glomerular layer.

Basal progenitor cells in the OE generate new OSNs throughout the life span of the mouse (for review, see Brann and Firestein, 2014). Although the dynamics of OE development and the targeting of OSN axons to the OB have been widely studied during early perinatal development, little was known of those events in the mature young adult. Here, we illustrate the dynamics of OSN neurogenesis and examine OSN behavior in a mature olfactory system. These data are fundamental to understanding the processes of adult neurogenesis and the incorporation of newly generated neurons into established circuits. Specifically, we provide critical information about the behavior of OSNs in adult stages that includes the following: the timeline of radial migration throughout the OE, molecular maturation, and axonal extension toward the OB, as summarized in Figure 11.

### Neurogenesis dynamics in the mature OE

In this study, using P25 mice we demonstrate that the ventral septum harbors significantly more newborn OSNs (approximately sixfold more) than the dorsal septum. This is consistent with our observation that the number of proliferative cells is higher in the ventral region compared with the dorsal region. Moreover, our data show that iOSNs in the ventral OE migrate faster through the OE (Fig. 4*B*). These results complement previous studies reporting a thinner layer of mOSNs in the ventrolateral zone IV of the OE, when compared with zone I (dorsal OE; Vedin et al., 2009). Given the higher proliferation rate and more rapid migration, our results are consistent with the hypothesis that OSNs generated in the ventral OE have a relatively shorter life span (Vedin et al., 2009).

Individual OSNs express only one OR gene, and each OR gene is confined to overlapping domains in the OE, which are summarized as zones (zones I, II, III, and IV). Thus, we cannot rule out that OSNs expressing different ORs undergo different proliferation rates. The ventral part of the septal wall that we analyzed corresponds to zones II and III, and the OSNs populating these zones express predominately class II genes (Miyamichi et al., 2005). In contrast, the dorsal septum, which we analyzed here, is considered zone I, and is populated predominately by class I and some class II OR genes (Miyamichi et al., 2005). Collectively, these data suggest that the proliferation rate in the adult OE could be influenced by the OR expressed. A differential onset is also consistent with the findings of previous studies showing that the first expression and subsequent expansion of individual OR expression is unique (Rodriguez-Gil et al., 2010), and of recent studies that demonstrate differential OSN proliferation in dorsal versus ventral OE (Eerdunfu et al., 2017).

### Maturation of OSNs

Our results suggest that a newborn OSN requires >24 h following cell division to differentiate into an iOSN, as measured by the onset of GAP 43 expression. This may reflect the time period necessary for progenitor cells to give rise to precursor cells (Neurogenin1^+^, NeuroD^+^). These precursor cells subsequently commit to a gene expression pattern that allows them to differentiate into the OSN (for review, see Suárez et al., 2012; Brann and Firestein, 2014). Previous studies reported that the presence of GAP 43 is transiently high in a temporal window between 3 and 9 d following injection of the thymidine analog EdU (Coleman et al., 2017), and between 5 and 10 d for Gγ8 following basal cell division (Savya et al., 2019), both of which are markers for iOSNs. Our results are overall in agreement with these previous findings; we show that GAP 43 is first expressed at DPI-Tx-3, peaks at DPI-Tx-5, maintains high expression through DPI-Tx-10, and decreases significantly afterward.

GAP 43 is implicated in the regulation of actin dynamics in growth cones, axonal growth (Williams et al., 2016), and synaptic plasticity in adult neurons (Holtmaat et al., 1997). GAP 43 overexpression is associated with increased axonal growth (Aigner et al., 1995; Leu et al., 2010; Donnelly et al., 2011, 2013) and axon sprouting and regeneration after injury (Grasselli et al., 2011; Allegra Mascaro et al., 2013). Correspondingly, a lack of GAP 43 is related to defects in neuronal development and axonal guidance (Strittmatter et al., 1995; Donovan et al., 2002; Shen et al., 2002; McIlvain et al., 2003). These data suggest that GAP 43 plays a key role in neuronal plasticity and growth. We show that in the OE the number of GAP 43^+^ OSNs remains high between 3 and 10 d following basal cell division. This time window of robust GAP 43 expression coincides with the extension process of their axons and navigation of the established olfactory nerve from the basal lamina of the OE to the OB, as our fate-mapping strategy shows.

To understand the ongoing innervation of the OB by adult-born OSN axons in the young adult, we addressed the following question: do OSNs require a similar timeframe to mature and innervate the OB regardless of the stage of development of the olfactory system? We demonstrate that OMP is expressed at DPI-B-8 (4.67% of BrdU^+^ OSNs express OMP) in P25 mice. Our data are consistent with previous studies that used different approaches to label cells at the time of neurogenesis and analyze OMP expression in OSNs of different ages and, in some cases, following OE injury. In concordance with our data, these alternate approaches also found that OSNs do not express OMP before 6–7 d following basal cell division, regardless of the age of the animal or the OE condition (Miragall and Monti Graziadei, 1982; Schwob et al., 1992; Kondo et al., 2010; Kikuta et al., 2015; Rodriguez-Gil et al., 2015; Savya et al., 2019). Our findings show that the number of OMP^+^ cells increases significantly after 8 d following basal cell division (Table 2, Fig. 4*E*), which corresponds temporally with axons reaching the surface of the OB and initial innervation of the glomerular neuropil (Fig. 10*C*,*D*). Earlier studies suggested that OSN synaptogenesis begins at the time of glomerular innervation (Farbman and Margolis, 1980), which would then correlate with the downregulation of GAP 43 and the upregulation of OMP. Addressing this more comprehensively with birthdate labeling of axons would be a timely and a more effective assessment of this hypothesis.

Of interest, prior work (Coleman et al., 2017) found that the percentage of EdU^+^ cells expressing OMP 5 d following EdU injections was slightly higher than what we observed in this work. The small discrepancy between our timelines may be accounted for by different quantitative analyses or animal housing conditions (Hinds et al., 1984; Mackay-Sim and Kittel, 1991; Holl, 2018). Moreover, as we report here and as others have noted (Vedin et al., 2009), the OE is a heterogeneous and asynchronous system with respect to neurogenesis and differentiation. Coleman et al. (2017) studied neurogenesis dynamics in areas containing OSNs expressing the olfactory receptor P2 (olfr17), while we included the entire septal OE. Although waves of proliferation or differentiation have not been described, we previously demonstrated that P2 is among the early-appearing ORs during embryonic development (Rodriguez-Gil et al., 2010), which is consistent with the notion that both the initial appearance and timeline to maturation may differ among subpopulations of OSNs.

AC3 expression is required for a canonical OSN to mature and reach the appropriate glomerulus, and to establish functional synaptic contacts. Alterations of the AC3 expression and cAMP pathway disturb axon convergence and the accurate structural organization of glomeruli (Imai et al., 2006, 2009; Chesler et al., 2007; Zou et al., 2007; Maritan and Monaco, 2009; Miller et al., 2010). Moreover, a decrease in the number of specific glomeruli and a corresponding decrease in OMP have also been reported in AC3 knock-out mice (Col et al., 2007). Since the dynamic timing of AC3 expression may impact axon targeting, we wanted to determine when P25-born OSNs express AC3 and how its expression is related to OMP. We show for the first time that AC3 expression occurs early after basal cell division, at DPI-B-3, with a considerable increase 5 d later that is synchronous with the initial expression of OMP. Here we demonstrate that, similar to what was shown in perinatal stages (Rodriguez-Gil et al., 2015), AC3 expression precedes OMP. Moreover, at DPI-B-10 and DPI-B-12, we found that most of the OMP^+^ adult-born OSNs are also AC3^+^ (Fig. 6*B*,*C4*, dotted circle, 6*D4*, dotted circle). We did find a small group of BrdU^+^/OMP^+^ OSNs that do not express AC3 (Fig. 6*C4*, dotted square, 6*E4*, arrow). It seems plausible that the small percentage of OMP^+^/AC3^−^ OSNs we reported here could belong to a subpopulation of noncanonical OSNs (Trpc2^+^ type B; Omura and Mombaerts, 2014), although further studies are needed. Members of the transduction cascade, including cAMP, are key in axonal convergence and glomerular formation (Imai et al., 2006; Chesler et al., 2007; Mobley et al., 2010). Here we show that AC3 is upregulated beginning at DPI-B-5 in young adult mice, when axons are located in the oONL before reaching the iONL. This suggests that AC3 expression might be critical for the final sorting of axons and convergence into the appropriate glomerulus.

The first evidence of differentiation among newborn cells in the OE was the onset of polarity that included the simultaneous extension of both an apical process, most likely fated to become the apical dendrite, and a basal process, most likely fated to become the axon. At later stages, growth cones were evident on both the apical and basal processes. The nascent apical dendrite extended rapidly, before the completion of the cell body radial migration to a central zone of the OE or the onset of OMP expression. The first dendritic knobs at the lumen of the OE were seen at 2 d following basal cell division. In some cases, rudimentary nascent cilia appear to be present but are not yet robustly expressed by 10 d following basal cell division (Fig. 8*D*). Prior work (Schwarzenbacher et al., 2005) describes cilia maturation during embryonic development, but the authors were not able to identify the ages of individual cells, as we did here. Their data show that, as it occurs for the maturation of different glomeruli, OR-specific populations exhibit different timing of ciliogenesis. However, they also demonstrated that as soon as ciliogenesis occurs, OR proteins appear to be relocated in the outgrowing cilia, and their presence decreases in the dendritic knob.

In summary, our data establish the following: (1) neurogenesis in the adolescent OE is regionally heterogeneous, with neurogenesis more robust in the ventral septum; (2) the radial migration of neuroblasts following basal cell division is regionally heterogeneous with neuroblast migration, which is more rapid in the ventral domain of the septum versus the dorsal domain; (3) the timeline of molecular maturation of OSNs (GAP 43 > AC3 > OMP) is similar to that seen in newborn mice; (4) following basal cell division, neuroblasts remain largely quiescent for 24 h until they begin to extend differentiating apical dendrites and basal axons; (5) within 48 h, many OSNs exhibit dendrites that have begun to extend toward the lumen, most of which reach the lumen by 72 h; (6) beginning at 48 h, the new axons have crossed the basal lamina to join existing axon fascicles; (7) the OSN axons cross the cribriform plate by 4 d post-tamoxifen injection and begin to course along the surface of the OB; and (8) the initial approach to innervate glomeruli is evident at 8–10 d post-tamoxifen injection, with axons within the glomerular neuropil by 10 d. These data provide an important new perspective on the maturation and incorporation of adult-born neurons into neural circuits and the ability of these cells to navigate previously established complex neuropil. We conclude that in young-adult stages, OSN molecular maturation and axon extension grossly recapitulate the mechanisms observed in the perinatal stages with a slightly different rhythm.

## Synthesis

Reviewing Editor: Julie Bakker, University of Liege Decisions are customarily a result of the Reviewing Editor and the peer reviewers coming together and discussing their recommendations until a consensus is reached. When revisions are invited, a fact-based synthesis statement explaining their decision and outlining what is needed to prepare a revision will be listed below. . Note: If this manuscript was transferred from JNeurosci and a decision was made to accept the manuscript without peer review, a brief statement to this effect will instead be what is listed below.

Both reviewers have read your manuscript with great interest but have some comments to have it improved. I agree with reviewer 1 that the double-labeling in Figure 5 is hard to see, in particularly regarding BrdU and OMP. Although it is mentioned in the method section that both male and female mice have been used in these experiments, there is no further mentioning of it in the results or the discussion. Was a sex comparison really been made, were the number of males and females used equivalent? Or was it all “at random”? Some explanation will be needed.

Reviewer 1:

Overall, this is an excellent manuscript, but in need of some revisions.

1st) I think the data in this manuscript should be compared to :

Schwarzenbacher K, Fleischer J, Breer H. Formation and maturation of olfactory cilia monitored by odorant receptor-specific antibodies. Histochem Cell Biol.2005 Jun;123(4-5):419-28.

In terms of when the OR comes on relative to the morphologies observed Schwazenbacher et al.

2nd) I think that this manuscript has two different kinds of DPI (BrdU and 4OH-Tx).

In this regard two things need to be fixed.

a) All DPI labels in ref BrdU should be married to a DPI-BrdU label (or maybe DPI-B) and

b) All DPI labels in ref to 4OH-Tx should be married to a DPI-4OH-Tx (or maybe DPI-Tx)

Furthermore, Figure 1 (dealing with controls for DPI-4OH-Tx) comes before Figures2-7 dealing with DPI-BrdU. This is very confusing. Authors should either add a separate Figure 1 DPI-BrdU or simply move Figure 1 between current Figure 7 and 8 where it belongs.

3rd) line 455: AC3 expression is required for a canonical OSN to mature, reach the appropriate glomerulus.

What is the evidence for lack of OMP expression in AC3 KOs? I am not aware that this specific analysis has been performed.

Also:

Dal Col, J. A., Matsuo, T., Storm, D. R. and Rodriguez, I. (2007). Adenylyl cyclasedependent

axonal targeting in the olfactory system. Development 134,

2481-2489

show glomerular formation in AC3 KO mice. This would seem to contradict line 455.

Please explain.

4th) Please mention in the Mat and Methods that ZsGreen is not an axonal marker, thus some axonal staining may be missing.

Minor Edits Below:

line 2: “begin”->begins

line 8: “cell basal”->basal cell

line 24: “the adult”->adult

line 24: “throw”->shed

line 46: remove extra space after “robust”

line 50: “the life”->life

line 68: “is”->are

line 70-72: reword sentence?

line 104: “in a presence of

both,”->in the presence of both

line 109: “the skin”->and the skin

line 125: insert comma after

“ZsGreen”

line 133: “z-stacks”->and z-stacks

line 147: “40x”->a 40x

line 161: “in”->in the

line 163: “in”->in the

line 185: “analyzed, aged”->analyzed: age {or reword}

line 227: “, OSN

apoptosis,”-> of OSNs

line 254: “in in”

line 296: “maker”->marker

line 298: “cell basal”->basal cell

line 325-327: unclear sentence

line 311-312: unclear sentence

line 341: “translocate”->translocates

line 346: “BrdU”-> the BrdU

line 378: “has”-> have

line 380-383: unclear sentence

line 429: add “of” after “regardless”

line 434: add “of” after “regardless”

line 444-445: unclear sentence

line 449-451: unclear what is P2, add description “odorant receptor P2 (olfr17)”

line 488: “mature”->adult

line 489: “recapitulate”->recapitulates

FIGURE 5:

-Put arrow on co-labeled cells in these figures

-It's very hard to distinguish cells that are BrdU+ and positive for marker at the same time in these images, yet counts were made and charts and percentages were drawn from these with lots of confidence. Discuss the criteria used to make a judgment call for a cell to be considered “co-labeled.” From the images, it seems arbitrary from certain cells pictured.

-In images 5D8 and 5D9, it is especially hard to see that these BrdU+ are the cells coexpressing OMP+, yet a very large percentage generated from these counts. (The Gap43 image doesn't look much different.)

-Why does BrdU sometimes appear to stain the nucleus, but other times forms a ring? Maybe mention something about this in the text?

FIGURE 7:

-Mention in the text what specific arrows point to. It's very hard to tell what is triple-labeled from these images. On page 13, paragraph lines 311-327, you make the claim that at DPI 12, 11% of BrdU+ cells are OMP+ but not AC3+. Can you mention in the text which figure contains these cells? Is it possible that this percentage is so large because of ambiguous visualization? You give a possible explanation of a non-canonical population in the discussion. A picture of the cell that's AC3-/OMP+ (or specific mention in the text) could be helpful.

Figure 8-10. Amazing images. Extremely informative to the field to see these axons in the midst of their journey, being correlated to a timeline. Very well done.

Reviewer 2:

eN-TNWR-0266-19X

Sequential maturation of olfactory sensory neurons in the mature olfactory epithelium

In this study, the authors tracked the maturation of olfactory sensory neurons (OSNs) in young adult mice at postnatal day (P) 25 (P25-born OSN). They found that the timeline of molecular maturation of P25-born OSN is GAP43>AC3>OMP, which is similar to that of P7-born OSN. In contrast, the robustness of neurogenesis in the adolescent OE is regionally heterogeneous with more in the ventral septum than dorsal septum; the difference was not observed in perinatal OE. Interestingly, the radial migration of P25-born OSNs is more rapid in the ventral septum than the dorsal domain. The authors also demonstrated the progressive extension of OSN axons toward the olfactory bulb (OB) after division in the adolescent OE.

Overall, the data in the paper are interesting since we could see the correlation between molecular maturation and axonal extension of OSNs in adolescent OE, which would add critical information to understand the homeostasis in the OE-OB network. I think the authors responded well to all the comments from the previous reviewers. The data shown in the current manuscript are clear and support the authors' conclusions.

Minor comments:

1. Figure 3F may need to be changed. The following description confused me, “However, in contrast to the BrdU data, we also found a significant main effect of time for Ki67+ (p-value < 0.01). Post hoc multiple pair-wise comparisons established that the number of proliferating neurons significantly decreased from DPI-3 to DPI-5 in the ventral septal OE (lines 211-214)”. I understand that it was likely due to a type 1 error of the statistical analysis. If so, I do not think that it is worth to present the number of Ki67+ cells at different DPI days (DPI-1, 3, 5, and 7) since the number is unlikely to significantly change during P25-P32. It may be better to combine the data shown in Figure 3F to show the difference in the proliferation rate between ventral and dorsal septum in the adolescent OE.

2. Figure 11: the coloring of OSNs at DPI-8, 10, and 12 may be misleading. Since all blue, maroon, and grey are in one OSN, it may mislead the readers into thinking that GAP43, AC3, and OMP are co-expressed in a single mature OSN. If I understand correctly, most of the mature OSNs expressing AC3 and OMP are GAP43-negative. If so, I would like to suggest to modify the illustration to reflect this point.
